# Gut Microbiome Differences in Rescued Common Kestrels (*Falco tinnunculus*) Before and After Captivity

**DOI:** 10.3389/fmicb.2022.858592

**Published:** 2022-06-20

**Authors:** Kangqing Zhang, Xinxiang Wang, Xiang Gong, Jinling Sui

**Affiliations:** School of Ecology and Nature Conservation, Beijing Forestry University, Beijing, China

**Keywords:** rescued common kestrel, captivity, gut microbiota, relative abundance, functional analysis

## Abstract

Gut microbes significantly impact animal health, yet research on the gut microbiota of most birds, especially raptors, is lacking. This study investigated the effects of dietary and environmental changes on the composition and abundance of gut microbiota in 17 rescued common kestrels (*Falco tinnunculus*) through 16S rRNA gene high-throughput sequencing of microorganisms in the feces of the birds. Firmicutes (relative abundance, 43.63%), Proteobacteria (37.26%), Actinobacteria (7.31%), and Bacteroidetes (5.48%) were the dominant phyla in the gut microbiota of the common kestrels. A comparison of the gut microbiota before and after captivity revealed that community composition and abundance of the common kestrel gut microbiota differed among different living conditions including diet and environment. At the phylum level, the abundance of Firmicutes was higher (*P* < 0.05), and that of Proteobacteria was lower (*P* < 0.05), after captivity (54.62 and 27.16%, respectively) compared with before captivity (33.67 and 46.41%, respectively), but no significant differences were found among other phyla. At the genus level, the abundance of *Lactobacillus* was higher (*P* < 0.05) after captivity (15.77%) compared with the abundance before captivity (5.02%). Hierarchical clustering and principal component analyses showed that common kestrels in different living conditions exhibited differences (*P* < 0.05) in gut microbiota at phylum and genus levels. Functional prediction of gene sequences using PICRUSt2 further revealed that pathways related to glucose metabolism and amino acid metabolism were enhanced (*P* < 0.05) after captivity. Collectively, the findings from this study demonstrated that the relative abundance of specific microbes in the gut of the rescued common kestrels either increased or decreased, and that dietary and environment changes might be the predominant factors affecting the gut microbiota of these birds during rescue or captivity.

## Introduction

Gut microbes play a crucial role in maintaining animal health, influencing physiological processes such as nutrient metabolism, vitamin synthesis, and immune function in the host (Round and Mazmanian, 2009; Hornef and Pabst, 2016; Ashrafian et al., 2019; Xu et al., 2020). Various studies have shown that gut microbes are extensively involved in the digestion of food in the gut, facilitating the breakdown of food into nutrients available to the host (Turnbaugh et al., 2006; Wu et al., 2010; Ley, 2016; Li et al., 2019). Carbohydrate metabolism in animals is facilitated by gut microbes, and many of the gut microbes that are commonly found can convert monosaccharides to short-chain fatty acids and transform pyruvate to lactate (Turnbaugh et al., 2009). Microorganisms in the well-developed crop of the hoatzins (*Opisthocomus hoazin*) can pre-ferment complex polymers in food into products that can be used by the host (Godoy-Vitorino et al., 2012), and can also degrade the toxic phenolics in plant food materials thus are involved in detoxification to a certain extent (Garcia-Amado et al., 2007).

The gut microbiota is also an indispensable part of the autoimmune function in animals, forming an immune barrier and preventing the invasion of foreign pathogens (Kamada et al., 2013; Yoon et al., 2021). Gut microbes coevolved with the host to form a symbiotic microbiota that maintained the health of the host, and these symbiotic bacteria have a close relationship with the host immune system. Rakoff-Nahoum et al. (2004) reported that the interaction of symbiotic bacteria and Toll-like receptors can maintain environmental stability in the animal gut. Meanwhile, Hammami et al. (2013) demonstrated that gut microbes can also maintain a stable environment through competitive rejection and production of antimicrobial compounds (e.g., bacteriocins and other toxins) to act against pathogens.

In addition to participating in host immune metabolism, microbes that colonize the gut are also influenced by host genetics (Lucas and Heeb, 2005; Whittaker et al., 2016; Song et al., 2020), feeding habits or diet (Preest et al., 2003; Blanco, 2014), developmental stages (González-Braojos et al., 2011; Escallón et al., 2019; Zhu et al., 2021), living conditions (Wu et al., 2021), and specific behaviors such as migration (Wienemann et al., 2011; Lewis et al., 2017; McCabe et al., 2020) and parasitism (Ruiz-Rodriguez et al., 2018). During the long-term co-evolution of host and gut microbes, a unique and stable gut microbial community or gut microbiota is formed in the host. Youngblut et al. (2019) studied the gut microbes of 128 wild animals and found that the gut microbes were predominantly composed of the Firmicutes, Proteobacteria, Actinobacteria, and Bacteroidetes, and that host diet and microbial phylogenetic relationships are crucial factors driving host-gut microbial variation. Similarly, a study of the gut microbes of 59 neotropical birds revealed that the composition of core microbes in the bird gut resembled that of previous studies (Grond et al., 2017; Huang et al., 2018; Kohl et al., 2019; Capunitan et al., 2020), with diet, different digestive tract regions, and habitats all influencing bird gut microbes (Preest et al., 2003; Blanco, 2014; Hird et al., 2015; Gongora et al., 2021).

*Ex situ* conservation is an important method for wildlife conservation, and many rare and endangered species are released back to nature after successful artificial rescue by zoos, rescue centers, and other institutions (Yu et al., 2015). Captive environments can alter animal behavior, affect normal physiological status, and in some cases reduce reproductive success rate (Amato et al., 2013; Amato et al., 2016; Tubbs et al., 2016). Numerous studies have analyzed the effects of captivity on wildlife gut microbes from a health management perspective (Kohl et al., 2014; Clayton et al., 2016). Most wild animals undergo significant compositional changes in their gut microbiota after captivity, with alterations in food and environment potentially being the predominant factors contributing to these changes (Li et al., 2017b; Gibson et al., 2019). In Oriental white stork (*Ciconia boyciana*), changes in food after captivity was an important factor leading to significant differences in species diversity and abundance of gut microbial communities (Wu et al., 2021). For birds, a shift in the survival environment is one factor that can rapidly alter microbial community composition (Xenoulis et al., 2010; Wienemann et al., 2011; Zhu et al., 2021). Despite active efforts to create a suitable environment for wildlife (enrichment) and feed a diet similar to that of the wild population during translocation and conservation, significant differences remain between the living environment and diet composition of wild and captive animals (Liu et al., 2021; Suzuki et al., 2021), causing microbial changes in the gut that impact the health status of the host. Therefore, investigating changes in gut microbiota composition in captive animals during rescue is essential to understand the health status of the animals and adaptation to artificial food supply and captive environments.

The common kestrel (*Falco tinnunculus*) is a Class II protected animal in China, as listed in the CITES Appendix II. This bird species has a wide distribution and usually inhabits low hills, farmland, and villages, and can also live in areas with intensive human activities (Charter et al., 2007). Common kestrels predominantly feed on small vertebrates such as mice, passerine birds, frogs, lizards, and snakes, as well as small insects such as locusts and crickets (Geng et al., 2009; Carrillo et al., 2017). Common kestrels—rescued for reasons such as weakness, fractures or poor flying ability—can carry a large number of microbes, including infectious pathogenic microbes that can damage their own health and be a source of transmission to other animals and even rescuers (Chu et al., 1976; Kwan et al., 2014; Boros et al., 2015; Guan et al., 2020). Rescued common kestrels are housed in human-built premises, which are different from their natural habitat. The food in captivity is mainly artificially bred mice, and this relatively homogeneous diet may cause stress to the common kestrels. Traditional rescue methods focus on the diagnosis and treatment of specific diseases, ignoring the impact of many gut microbes on the host. Focusing on differences in gut microbiota between wild and captive conditions and analyzing the possible role of these differences in nutrient absorption and immune defense might improve the adaptive ability of individual rescued common kestrels to return to nature from captivity. In addition, the samples in previous studies in exploring the effects of wild and captive environments on gut microbiota were mostly from different populations, and it was inevitable that individual differences would affect the accuracy of the results (Gibson et al., 2019; Liu et al., 2021; Suzuki et al., 2021). The current study utilized 16S rRNA gene high-throughput sequencing technology to analyze the gut microbiota of common kestrels rescued by Beijing Wildlife Rescue Center before and after captivity, with the aim of exploring the changes in composition and diversity of the gut microbiota during rescue or captivity. Findings from the study provide a scientific basis for the diagnosis of gut diseases of rescued common kestrels and lay a foundation for the improvement of health management methods in captivity to facilitate the return of these birds to nature.

## Materials and Methods

### Animals

Animals used in this study were 17 common kestrels rescued by the Beijing Wildlife Rescue Center from May to June in 2021. The common kestrels were divided into three different age groups according to the status of the molting cycle and the shape and wear of the feathers (Supplementary Table 1), of which five were adults, six were subadults, and six were juveniles. The sex of each common kestrel was identified by PCR amplification of DNA from the back feathers using the universal primers 2550F (5′-GTTACTGATTCGTCTACGAGA-3′) and 2718R (5′-ATTGAAATGATCCAGTGCTTG-3′) (Cakmak et al., 2017) (Supplementary Table 1), and eight males and nine females were identified. All individuals showed no obvious signs of disease after veterinary examination, so were not treated with drugs, and were housed individually in isolation cages and fed daily with freshwater, mice, and/or beef.

### Collection and Storage of Samples

Sampling was conducted at two stages: firstly, samples were collected immediately after the rescue (i.e., before captivity); secondly, samples were collected immediately before the release of the recovered common kestrels (i.e., after captivity). For sampling, each rescued common kestrel was marked and placed into a shaded cage lined with sterile white paper. Immediately after the individual defecated, fecal samples were collected with sterile cotton swabs and placed into centrifugal tubes. All of the above steps were done under the premise of ensuring that the samples collected were not contaminated by other environmental factors including the sampler. The tubes were labeled and stored in at −80°C until further use. No less than 2 g fresh feces were collected in each centrifuge tube. Sampling avoided uric acid as much as possible to ensure that sequencing was not affected. After the rescue, quiet common kestrel individuals were selected for sampling to avoid or reduce the possible adverse effects of a strong stress reaction. A total of 34 common kestrel fecal samples were collected in this study, including 17 collected before captivity and 17 after captivity for 4–14 days (Supplementary Table 2).

### DNA Extraction

Bacterial DNA was extracted from feces using the QIAamp DNA Stool Mini Kit (QIAGEN, Hilden, Germany) and the concentration and integrity of the extracted DNA were determined by NanoDrop and agarose gel electrophoresis, respectively. Samples with a concentration greater than 5 ng/μL and a distinct main band on the agarose gel were selected for PCR amplification with universal primers 338F (5′-ACTCCTACGGGAGGCAGCA-3′) and 806R (5′-GGACTACHVGGGTWTCTAAT-3′). The 10 μL reaction system was composed of 5 ng DNA, 0.3 μL each primer (10 μM), 5 μL KOD FX Neo Buffer, 2 μL dNTPs (2 mM), 0.2 μL PCR enzyme (KOD FX Neo), and ddH_2_O to 10 μL. PCR cycling conditions included a pre-denaturation step at 95°C for 5 min, 25 cycles of denaturation at 95°C for 30 s, annealing at 50°C for 30 s, and extension at 72°C for 40 s, followed by an extension step at 72°C for 7 min and then termination at 4°C. The amplified PCR products were detected by electrophoresis using agarose gels at a concentration of 1.8%, and samples with distinct main bands were selected for building the database. The database was then checked by the Qsep-400 method and quantified by Qubit 3.0. Double-end sequencing was performed on the Illumina Novaseq 6000 platform after the library was qualified quantitatively (i.e., concentration ≥ 1 ng/μL, fragment center value 430–530 bp, average value 420–580 bp, peak shape normally distributed, and the fragment being single without heteropeaks).

### Sequencing Data Processing

Raw reads obtained from sequencing were filtered using Trimmomatic v0.33 software, then the primer sequences were identified and removed by Cutadapt 1.9.1 software to obtain high-quality clean reads. Sequences from each sample were spliced and then length filtered by Usearch v10 software, and chimeric sequences were identified and removed by UCHIME v4.2 software to obtain the final valid data (effective reads). The sequences were clustered at the 97% similarity level using Usearch v10 software to obtain operational taxonomic units (OTUs). Raw sequences obtained in this study are available through the National Center for Biotechnology Information (NCBI) database (accession number PRJNA797889).

### Operational Taxonomic Unit Sequence Annotation and Taxonomic Analysis

Taxonomic analysis of OTU sequences was performed using a plain Bayesian classifier with SILVA as the reference database. This generated taxonomic information on the species corresponding to each feature and then allowed analysis of microbial community composition at different levels, including phylum, class, order, family, genus, and species. Species abundance lists at different taxonomic levels were generated using QIIME software, and then the community structure at each taxonomic level was mapped using R software.

### Statistical Analysis

Species richness and diversity indices, including ACE, Chao1, Shannon, and Simpson indices, were calculated using the vegan package (Dixon, 2003) in R software (3.6.3) and were used to analyze species diversity and complexity. Weighted UniFrac distances between samples were calculated using the phyloseq package, then differences in gut microbial communities between the two groups of samples at the phylum and genus levels were assessed separately using NMDS analysis, and the significance of differences between the two groups was tested by the permutation multivariate analysis of variance (PERMANOVA). Hierarchical clustering among samples was obtained by using the unweighted pair group method with the arithmetic means (UPGMA) function of the vegan package. Heatmaps, dendrograms, venn diagram, and between-group analysis of variance maps were generated using R software and categorized by the online linear discriminant analysis effect size (LEfSe) website.^1^ A microbial co-occurrence network was constructed based on the Spearman correlation coefficients between the relative abundances of genera, and the association network was visualized using Gephi 0.9.1 software. The function of each OTU was predicted using Phylogenetic Investigation of Communities by Reconstruction of Unobserved States (PICRUSt2) (Douglas et al., 2020). The predicted functions (KOs) were then collapsed into hierarchical KEGG (Kyoto Encyclopedia of Genes and Genomes) pathways in the PICRUSt2 pipeline. Annotation of the function of gut microbes of common kestrels was performed according to the KEGG database (Kanehisa et al., 2014). A correlation heatmap between microbial taxa and three levels of metabolic pathways was plotted using the pheatmap function based on the Pearson correlation coefficient between the relative abundance of bacterial populations and the relative abundance of predicted pathways. Relative abundances were expressed as Mean ± SE. Differences between samples before and after captivity were tested for significance. Paired sample *t*-test was used for data satisfying the conditions of normality; otherwise, the paired Mann-Whitney *U*-test was used. *P* values were corrected by false discovery rate (FDR), and *P* < 0.05 was considered statistically significant.

## Results

### Validation of 16S rRNA Gene Sequencing of Common Kestrel Feces Samples

All samples were subjected to 16S rRNA gene sequencing, and after quality control, a total of 3,139,268 high-quality sequences were obtained, of which 3,126,045 were valid sequences, accounting for 99.58% of the total high-quality sequences. An average of 91,943 valid sequences were obtained for each sample, indicating that the sequencing data were sufficient to cover most of the gut microbes. The dilution curve (Supplementary Figure 1) showed that the number of OTUs increased rapidly with a linear growth pattern when the sequencing volume was small. However, when the sequencing volume was larger, the increase rate of the OTUs gradually decreased and finally leveled off, indicating that the amount of sequencing data obtained was sufficient to reflect the species diversity in the samples and thus ensured the reliability of the subsequent analyses.

### Gut Microbe Composition of Common Kestrels

**FIGURE 1:**
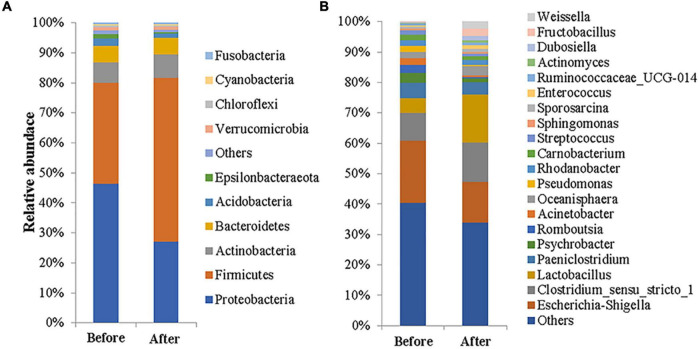
Gut microbial compositions of the common kestrel before and after captivity. The compositions are presented as relative abundances at the phylum **(A)** and genus **(B)** levels.

A total of 23 phyla, 51 classes, 124 orders, 222 families, 484 genera, and 561 species of microbes were detected among the 34 feces samples. At the phylum level, there were six phyla with a relative abundance higher than 1% (Supplementary Figure 2), namely Firmicutes (43.63%), Proteobacteria (37.26%), Actinobacteria (7.31%), Bacteroidetes (5.48%), Acidobacteria (2.03%), and Verrucomicrobia (1.04%), which accounted for 96.75% of the total bacteria, and the sum of the two most abundant phyla was 80.89%. Before captivity (Figure 1A), there were seven phyla with a relative abundance greater than 1%, including Proteobacteria (46.41%), Firmicutes (33.67%), Actinobacteria (6.80%), Bacteroidetes (5.58%), Acidobacteria (2.47%), Epsilonbacteraeota (1.41%), and Verrucomicrobia (1.16%), which accounted for 97.50% of the total bacteria, and the sum of the two most abundant phyla was 80.08%. In contrast, only five phyla with a relative abundance of more than 1% were found after captivity, including Firmicutes (54.62%), Aspergillus (27.16%), Actinomycetes (7.86%), Bacteroidetes (5.36%), and Acidobacteria (1.56%) (Figure 1A); these phyla accounted for 96.56% of the total, and the sum of the two most abundant phyla was 81.78%.

At the genus level (Figure 1B), the nine genera with a relative abundance exceeding 2% before captivity were *Escherichia-Shigella* (20.45%), *Clostridium_sensu_stricto_1* (9.08%), *uncultured_bacterium_ f_Enterobacteriaceae* (6.59%), *Lactobacillus* (5.02%), *Paeniclostridium* (4.91%), *Psychrobacter* (3.39%), *Romboutsia* (2.55%), *Acinetobacter* (2.27%), and *Oceanisphaera* (2.07%). These genera accounted for 59.12% of the total bacteria, but only one genus, *Escherichia-Shigella*, had a relative abundance greater than 10%. In addition, there were seven genera with a relative abundance above 1% (totaling 10.71%). In contrast, the nine genera whose relative abundance exceeded 2% after captivity were *Lactobacillus* (15.77%), *Escherichia-Shigella* (13.43%), *Clostridium_sensu_stricto_1* (12.98%), *Paeniclostridium* (4.06%), *uncultured_bacterium_ f_Actinomycetaceae* (3.05%), *Oceanisphaera* (2.94%), *uncultured_bacterium_f_Muribaculaceae* (2.57%), *Fructobacillus* (2.44%), and *Weissella* (2.09%). These genera accounted for 59.32% of the total, of which three genera—*Lactobacillus*, *Escherichia-Shigella*, and *Clostridium_sensu_stricto_1*—had relative abundances exceeding 10%. In addition, there were six genera with a relative abundance of more than 1% after captivity, accounting for 7.83% of the total.

### Differences in Gut Microbes of Common Kestrels Before and After Captivity

#### Alpha- and Beta-Diversity Analyses

For alpha-diversity analysis, the abundance and diversity of the gut microbes of common kestrels under different living conditions were assessed using ACE, Chao1, Simpson, and Shannon indices (Table 1). The ACE index was higher after captivity (640.66) compared with before captivity (636.19), while the Chao1 index was higher before captivity (622.85) compared with after captivity (607.51). Simpson and Shannon indices were both higher after captivity (0.84 and 4.78, respectively) compared with before captivity (0.81 and 4.55, respectively). A comparison of the differences in richness and diversity indices between the two groups of feces samples before and after captivity showed that the ACE (Wilcoxon test: *W* = 80, *P* = 0.890), Chao1 (Wilcoxon test: *W* = 62, *P* = 0.517), Simpson (Wilcoxon test: *W* = 94, *P* = 0.431), and Shannon (Wilcoxon test: *W* = 85, *P* = 0.712) indices were all not significantly different at the *P* > 0.05 level. The effects of captive environment on the alpha diversity of gut microbes in different age and sex groups were then examined. The results showed that there was no significant effect on alpha diversity in the gut of common kestrel with different genders and ages (*P* > 0.05, Supplementary Table 5).

**TABLE 1:** Comparisons of gut microbial abundance and diversity indices between the common kestrels before and after captivity.

	ACE	Chao1	Simpson	Shannon	Coverage
Before captivity	636.19 ± 35.38	622.85 ± 30.53	0.81 ± 0.04	4.55 ± 0.47	0.9989
After captivity	640.66 ± 37.80	607.51 ± 27.81	0.84 ± 0.03	4.78 ± 0.32	0.9989
*P* values	0.89	0.52	0.71	0.43	

**FIGURE 2:**
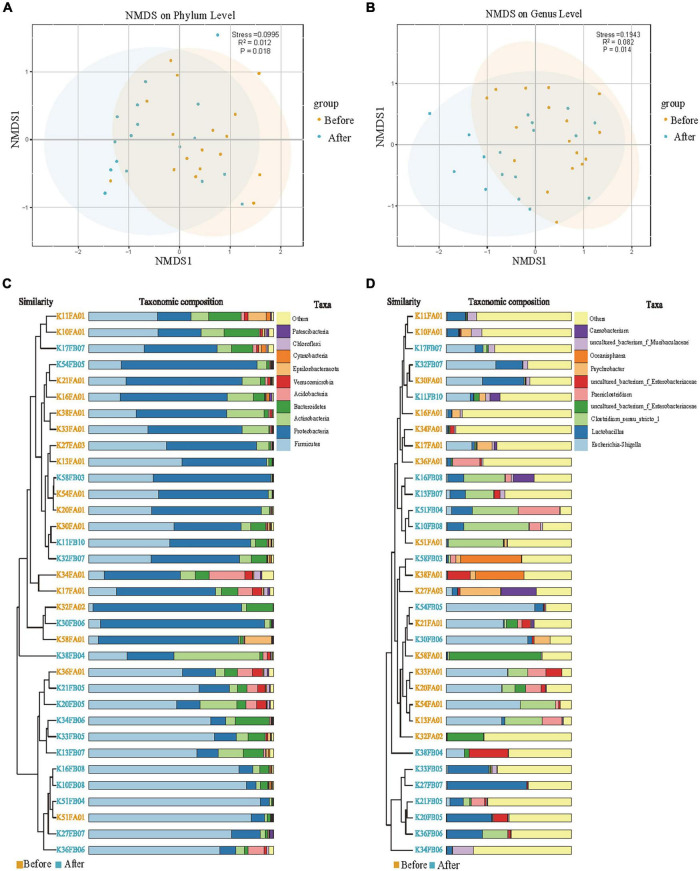
Beta-diversity analysis of gut microorganisms between the rescued common kestrels before and after captivity. **(A,B)** NMDS analyses based on weighted UniFrac distances at phylum and genus levels, respectively. **(C,D)** UPGMA clustering at phylum and genus levels, respectively.

NMDS analysis and UPGMA clustering analysis were used to evaluate the beta diversity of fecal microbial composition (Figure 2). NMDS analysis based on the weighted Unifrac distances revealed significant separation (*P* < 0.05) in the composition of gut microbes between the before and after captivity groups at both the phylum (PERMANOVA test: *R*^2^ = 0.112, *P* = 0.018; Figure 2A) and genus (PERMANOVA test: *R*^2^ = 0.082, *P* = 0.014; Figure 2B) levels. Similarly, UPGMA clustering analysis based on the weighted Unifrac distances showed a higher similarity of microbial communities within groups compared with between groups at both the phylum and the genus levels (Figures 2C,D). The effects of captivity environment on the beta diversity of gut microbes in different age and sex groups were subsequently analyzed. The results showed that the captive environment had a significant effect on the beta diversity at the phylum level in the gut of male common kestrels (*R*^2^ = 0.225, *P* = 0.020), but had no significant effect in female common kestrels (*P* > 0.05, Supplementary Table 6). And there was no significant difference in beta diversity among individuals of different age groups before and after captivity (*P* > 0.05, Supplementary Table 6).

#### Comparison of Specific Microbes Between the Rescued Common Kestrels Before and After Captivity

**FIGURE 3:**
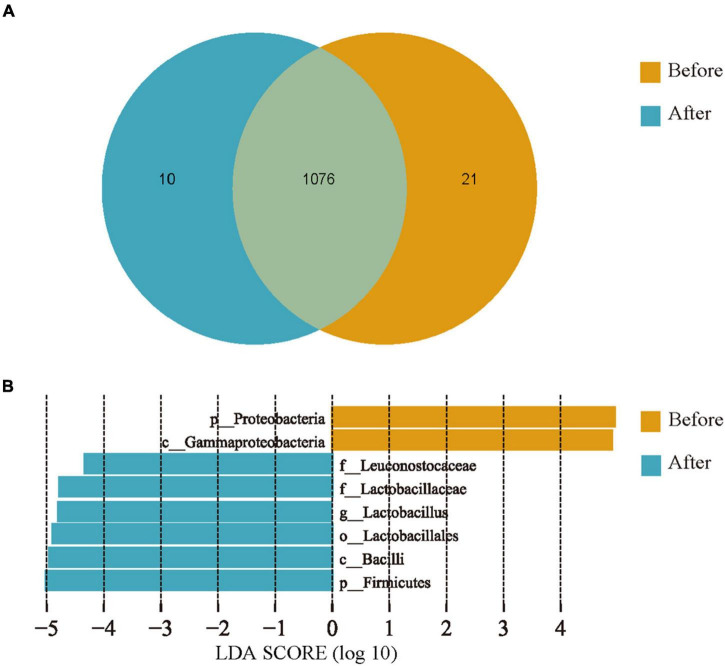
Comparison of the unique bacteria in gut microbiota of the common kestrels before and after captivity. **(A)** Venn diagram showing numbers of common and unique OTUs. **(B)** LEfSe analysis at the genus level.

Operational taxonomic unit clustering of non-duplicate sequences was performed according to 97% similarity, and a total of 1,107 OTUs were selected as representative sequences. Among these representative sequences, there were 1,088 and 1,086 OTUs obtained before and after captivity, respectively, with 1,076 OTUs shared between both groups, 10 OTUs uniquely detected after captivity, and 21 OTUs only detected before captivity (Figure 3A).

LEfSe analysis, under the threshold of LDA > 4.0, revealed significant differences between the common kestrels before and after captivity (Figure 3B), of which the relative abundances of Proteobacteria and Gamma-proteobacteria were higher (*P* < 0.05) before captivity compared with after captivity. In contrast, the relative abundances of Bacilli, Lactobacillaceae, and Leuconostocaceae were higher (*P* < 0.05) after captivity compared with before captivity.

**FIGURE 4:**
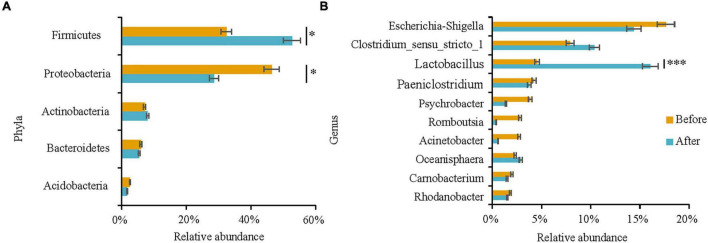
Comparison of differences in the main compositions of gut microbiota of the common kestrels before and after captivity. Differences in relative abundance among the top five core phyla **(A)** and the top 10 core genera **(B)** were analyzed. * and ^***^ indicate significant differences at *P* < 0.05 and *P* < 0.001, respectively.

The top five phyla (i.e., the sum of relative abundances of the top five phyla, >90%) and the top 10 genera (i.e., the sum of relative abundances of the top 10 genera, >50%) were selected for comparison of differences between the two groups of fecal bacteria. At the phylum level, Firmicutes was higher after captivity compared with before captivity (*t*-test: *t* = 2.515, *df* = 30.774, *P* = 0.017), while Proteobacteria was higher before captivity compared with after captivity (Wilcoxon test: *W* = 77, *P* = 0.020). The relative abundances of Actinobacteria (Wilcoxon test: *W* = 131, *P* = 0.658), Bacteroidetes (Wilcoxon test: *W* = 143, *P* = 0.973), and Acidobacteria (Wilcoxon test: *W* = 137, *P* = 0.812) were not significantly different between the two groups of feces (Figure 4A). At the genus level, *Escherichia-Shigella* (Wilcoxon test: *W* = 134, *P* = 0.734), *Clostridium_sensu_stricto_1* (Wilcoxon test: *W* = 148, *P* = 0.919), *Paeniclostridium* (Wilcoxon test: *W* = 133, *P* = 0.708), *Oceanisphaera* (Wilcoxon test: *W* = 144, *P* = 1), *Psychrobacter* (Wilcoxon test: *W* = 105, *P* = 0.182), *Rhodanobacter* (Wilcoxon test: *W* = 152.5, *P* = 0.796), *Carnobacterium* (Wilcoxon test: *W* = 142, *P* = 0.945), *Romboutsia* (Wilcoxon test: *W* = 138.5, *P* = 0.850), and *Acinetobacter* (Wilcoxon test: *W* = 144, *P* = 1) did not differ among the two groups of feces. In contrast, the relative abundance of *Lactobacillus* was higher after captivity compared with before captivity (Wilcoxon test: *W* = 236, *P* = 0.001) (Figure 4B). Next, the effects of captivity environment on the abundance of dominant phylum and genus in different age and sex groups were analyzed. The abundance of Proteobacteria (*t* = −2.476, *df* = 13.969, *P* = 0.027) and *Lactobacillus* (*t* = 2.409, *df* = 11.742, *P* = 0.033) in male common kestrels was significantly different before and after captivity. The abundance of dominant phylum and genus was not significantly affected by captivity environment in females and individuals of different ages (*P* > 0.05, Supplementary Table 7).

#### Gut Microbiota Association Network Analysis and Functional Annotation

**FIGURE 5:**
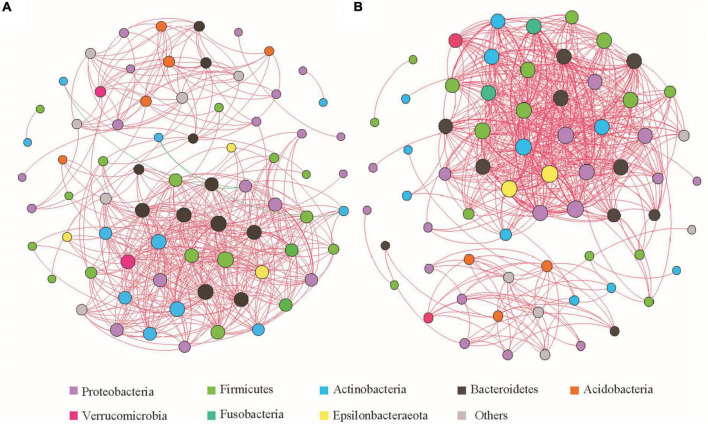
Spearman correlation network analysis of gut microbiota of the common kestrels before and after captivity at the genus level. **(A**,**B)** Correlation networks before and after captivity, respectively, with the node size reflecting the number of connections and the color of the node corresponding to the phyla as listed. Red and green lines represent positive and negative correlations, respectively, and the thickness of the line indicates the strength of the correlation.

The top 100 genera in terms of relative abundance (Supplementary Table 3) were selected and their Spearman correlation coefficients were calculated and used to analyze the correlation between the gut microbiota in the two groups of feces (Figure 5). The degree of microbial co-occurrence networks of the two groups conformed to a power-law distribution (Before captivity: *y* = 7.464x^–0^.^481^, *R*^2^ = 0.467, *P* = 0.001; After captivity: *y* = 6.597x^–0^.^467^, *R*^2^ = 0.224, *P* = 0.012). Genera with highly correlated relative abundance changes clustered in connected modules. The group before captivity formed seven modules (Figure 5A), with 477 links between 100 nodes (mean degree 13.068, mean path length 2.694, and mean clustering coefficient 0.717), while the group after captivity formed six modules (Figure 5B), including 547 links between 100 nodes (mean degree 16.328, mean path length 3.064, and mean clustering coefficient 0.76).

Before captivity, most of the genera in which microbial communities tended to co-occur (positively correlated) belonged to the phyla Firmicutes, Proteobacteria, Bacteroidetes, and Actinobacteria. In contrast, the genus *Brevundimonas* belonging to the phylum Proteobacteria, and the genus *Rothia* of the Actinobacteria, showed co-rejection (negative correlation). After captivity, all microbial communities tended to co-occur, but compared with those before captivity, the co-occurrence network was more complex in the phyla Firmicutes, Proteobacteria, Bacteroidetes, and Actinobacteria, as well as in some genera of the phyla Verrucomicrobia and Epsilonbacteraeota.

### Functional Annotation of Gut Microbes

The functional potential of the microbial communities present among common kestrel gut microbes was predicted by PICRUSt2 and involved functional pathways such as “metabolic pathways,” “biosynthesis of secondary metabolites,” “biosynthesis of antibiotics,” “microbial metabolism in diverse environments,” “biosynthesis of amino acids,” “ABC transporters,” “carbon metabolism,” and “two-component system” (Supplementary Table 4).

The top 30 functional pathways in terms of relative abundance were analyzed and significant differences (*P* < 0.05) were found between the common kestrels before and after captivity in the pathways of “microbial metabolism in diverse environments,” “carbon metabolism,” “purine metabolism,” “ribosome,” “pyrimidine metabolism,” “amino sugar and nucleotide sugar metabolism,” “glycolysis/gluconeogenesis,” “aminoacyl tRNA biosynthesis,” “alanine/aspartate and glutamate metabolism,” “homologous recombination,” “glyoxylate and dicarboxylate metabolism,” “mismatch repair,” and “peptidoglycan biosynthesis” (Supplementary Table 4).

**FIGURE 6:**
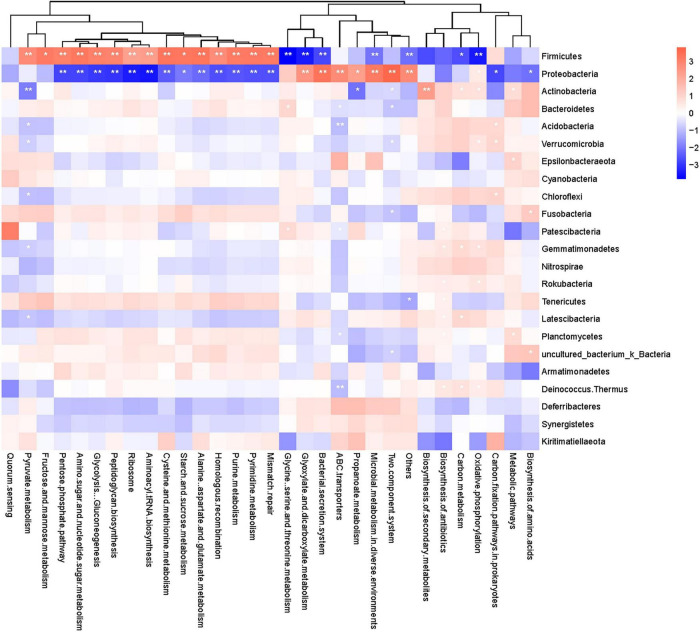
Heatmap of correlation between metabolic pathways and gut microbiota of the common kestrels at the phylum level. The horizontal axis shows metabolic pathways, and the vertical axis shows different phyla. Red and blue indicate positive and negative correlations, respectively, and the deeper the color, the stronger the correlation. *and ** indicate significant differences at *P* < 0.05 and *P* < 0.01, respectively.

A heatmap of correlations between the different phyla and the top 30 functional pathways (based on relative abundance) (Figure 6) showed that Firmicutes were positively correlated with glucose metabolism- and amino acid metabolism-related pathways, including “pentose phosphate pathway,” “amino sugar and nucleotide sugar metabolism,” “glycolysis/gluconeogenesis,” “peptidoglycan biosynthesis,” “cysteine and methionine metabolism,” and “starch and sucrose metabolism,” while Proteobacteria were negatively correlated with these pathways. In contrast, Firmicutes were negatively correlated with pathways related to “glyoxylate and dicarboxylate metabolism,” “bacterial secretion system,” and “glycine, serine and threonine metabolism,” whereas Proteobacteria were positively correlated with these pathways.

## Discussion

The gut microbiota of vertebrates is closely related to the health, growth, and development of the host (Kim et al., 2014), thus the study of bird gut microbiota has become an important element of wild bird conservation research. The current study compared the gut microbial compositions of common kestrels under two different living conditions (i.e., before and after captivity), and showed that the gut microbiota composition of the wild common kestrels changed to some extent during rescue.

### Changes of Dominant Gut Bacteria in the Rescued Common Kestrels During Artificial Feeding

Members of the phylum Proteobacteria are mostly pathogenic and usually associated with gut ecological imbalance, metabolic disorders, and immune dysregulation (Colston and Jackson, 2016), whereas members of the Firmicutes play important roles in the metabolism, digestion, and absorption of proteins and other nutrients, and participate in the synthesis of digestive enzymes to assist the digestion and absorption of nutrients by the host. The Firmicutes can also produce a large amount of butyrate, which improves insulin sensitivity, regulates energy metabolism, and enhances leptin gene expression (Grond et al., 2018). In the current study, the highest abundance of Proteobacteria (46.41% relative abundance) was found before captivity, followed by Firmicutes (33.67%), while the highest abundance of Firmicutes (54.62%) was found after captivity, followed by Proteobacteria (27.16%) (Supplementary Figure 2). This was consistent with the study of Zhou et al. (2020) but different from that of other wild raptors reported by Oliveira et al. (2020). Similar studies conducted in other bird species, such as the bar-headed goose (Wang et al., 2017) and the oriental stork (Wu et al., 2021), found that Firmicutes was the dominant species in both wild and caged populations, but the relative abundance of this phyla was lower in the caged population compared with the wild population. This may be due to differences in species or in the environment and diet between wild and caged common kestrels. Under wild conditions, the diet of the common kestrels include rodents, small reptiles, and other species, and is more diverse and of complex origin, with many wild animals also carrying pathogenic microorganisms (Bevins et al., 2016; Li et al., 2017a). Such pathogenic microorganisms acquired through the diet will inevitably increase the species and number of pathogenic microorganisms in the gut of wild common kestrels. In contrast, common kestrels in captivity were predominantly fed on artificially bred mice or beef, which had a single food source and species and carried fewer microorganisms, thus resulting in a higher abundance of the phylum Firmicutes and a lower abundance of the phylum Proteobacteria in the gut of the caged common kestrels.

Genera such as *Escherichia-Shigella*, *Acinetobacter*, *Pseudomonas*, *Psychrobacter*, and *Lactobacillus* are widely present in the gut of wild birds (Hird et al., 2015; Xie et al., 2016). The present study showed that the genera with high relative abundance before captivity, including *Escherichia-Shigella*, *Psychrobacter*, *Acinetobacter*, and *Oceanisphaera*, were predominantly from the phylum Proteobacteria (Figure 1) and were mostly opportunistic pathogens (Maria et al., 2016; Castano-Rodriguez et al., 2018; Liu et al., 2020; Zhang et al., 2021). In contrast, the genera with higher relative abundance after captivity, including *Lactobacillus*, *Clostridium_sensu_stricto_1*, *Paeniclostridium*, and *Fructobacillus*, were predominantly from the phylum Firmicutes (Figure 1), and were mostly associated with nutrient metabolism. Broadfield et al. (2021) recently reported that fat-induced glucose metabolism was enhanced in the liver of mice fed a high-fat diet and that with this type of high-fat diet, ^13^C markers in the liver were mostly enriched in glucose molecules among the glycolytic metabolites and the metabolites of many branching pathways such as the pentose phosphate pathway and serine biosynthesis pathway. Xenoulis et al. (2010) reported that *Lactobacillus* were the major gut microbes in caged birds, while Magnusson et al. (2015) demonstrated that a significant increase in the number of *Lactobacillus* in the gut of mice was related to their high-sugar diet. In the current study, the abundance of *Lactobacillus* in the gut microbiome of common kestrels after captivity were significantly higher compared with that before captivity (Figure 4), which may contribute to the digestion and absorption of high-fat and high-sugar foods in the caged kestrels. Meanwhile, since the relative abundance of *Lactobacillus* in males was significantly different before and after captivity (Supplementary Table 7), it is speculated that males were probably more sensitive to the captive environment and food changes.

LEfSe analysis also showed that the common kestrels under different living conditions had distinct characteristic taxa among their gut microbes. A total of eight taxonomic units (from phylum to genus) with LDA thresholds > 4.0 were identified in both groups, which were consistent with the findings obtained from other analytical methods in this study, i.e., the Proteobacteria were characteristic before captivity whereas the Firmicutes were characteristic after captivity (Figure 3B).

### Gut Microbial Diversity Characteristics of the Rescued Common Kestrels During Captivity

Alpha-diversity analysis showed that the cage environment had no significant effect on the abundance and diversity of the gut microbes of the common kestrels (Table 1), but NMDS diversity analysis based on weighted Unifrac distances showed a certain degree of relative separation between groups at both the phylum and genus levels before and after captivity. Meanwhile, because the captive environment has a significant effect on the beta diversity of males at the phylum level (Supplementary Table 6), therefore males were probably more sensitive to the changes of environment. UPGMA clustering analysis based on weighted Unifrac distances revealed that, at the phylum level, most individuals exhibited higher intra-group similarity compared with inter-group similarity. There were also a few individuals whose microbial composition after captivity was more similar to that before captivity, for example, some individuals retained a high abundance of the phylum Proteobacteria after captivity. This phenomenon may be due to the short duration of caging (Supplementary Table 2) and the limited influence of the environment, which was not yet sufficient to alter the community composition of the gut microbes. OTU clustering analysis showed a higher number of common OTUs before and after captivity and a lower number of OTUs that were unique to each of the two groups of feces samples (Figure 3A). The difference between individuals before captivity, which had a high abundance of Proteobacteria, and those after captivity, which had a high abundance of Firmicutes (Figure 1), was mainly due to differences in the relative abundance of Proteobacteria and Firmicutes, and not caused by phyla that were unique between the two groups.

### Changes in Gut Microbial Interactions During Captivity of the Rescued Common Kestrels

Analysis of association networks among microbes can provide a basis for the composition of microbial communities, and the study of patterns of interactions between genera that are co-existing or mutually exclusive can lead to inferences on whether different microbes are in a “cooperative” or “competitive” relationship. When two species are strongly related under the same environmental conditions, it means that the two species have a certain degree of ecological niche overlap, and co-exclusion may be caused by competition or niche differentiation (Olesen et al., 2007). In the current study, the correlation between microbes in the gut of the common kestrels was complex. Before captivity, potentially pathogenic organisms associated with intestinal diseases (e.g., *Desulfovibrio*, *Ochrobactrum*, and *Neisseria*) were positively correlated with beneficial bacteria (e.g., *Alistipes*, *Lactobacillus*, *Akkermansia*, and *Parabacteroides*) (Supplementary Table 3), suggesting that pathogenic bacteria and beneficial bacteria may occupy similar ecological niches in the gut of individuals living in the wild. The genus *Brevundimonas* of the phylum Proteobacteria was negatively correlated with some genera of the phylum Actinobacteria, further suggesting that the collaborative and competitive effects of the bacteria work together to maintain the stability of the gut microbiota in the common kestrels. The co-occurrence network of bacterial communities in the gut of the common kestrels after captivity was more complex compared with those obtained before captivity. Genera associated with fat metabolism (e.g., *Eubacterium coprostanoligenes* group, *Lactobacillus*, and *Parabacteroides*) formed the core nodes of the association network after captivity (Figure 5 and Supplementary Table 3), which reflected that the fat content in the food of the caged common kestrels might be higher than that of the same individuals living in the wild. Interactions between bacteria are clearly instrumental in maintaining the stability of the microbial community structure of caged common kestrels digesting higher fats.

### Adaptation of Gut Microbial Functional Groups to Environmental Changes in the Rescued Common Kestrels

The metabolic pathways of functional genes of the common kestrel gut microbes predicted using the PICRUSt2 algorithm are consistent with those predicted for metabolic functions of gut microbes in other wildlife (Jiang et al., 2021). Most functions were associated with carbohydrate metabolism, amino acid metabolism, nucleotide metabolism, and energy metabolism (Supplementary Table 4), suggesting that the gut microflora is important in the construction of metabolic capacity of the host. The current study also revealed significant changes in metabolic pathways such as carbon metabolism, amino sugar and nucleotide sugar metabolism, glycolysis/gluconeogenesis, polysaccharide biosynthesis and metabolism, and alanine, aspartic acid, and glutamate metabolism before and after captivity (Supplementary Table 4). Furthermore, the correlation heatmap between the phyla and metabolic pathways showed that the enhancement of sugar metabolism and amino acid metabolic pathways after captivity was significantly and positively correlated with the increase in abundance of Firmicutes (Figure 6). A high-fat and high-protein diet was previously shown to increase abundance of the Firmicutes and Proteobacteria (Cani et al., 2007; Murphy et al., 2010), implying that the enhanced glucose metabolism and amino acid metabolic pathways of the gut flora after captivity may be related to the feeding of a high-fat and high-protein diet. Although the PICRUSt2 algorithm contains a larger gene family and reference genomic database with higher accuracy than prior methods (Douglas et al., 2020), it does not represent a 100% correlation between the predicted genes and the true metabolic pathways of the population. The gut microbial community of the common kestrels was complex and may contain many additional undiscovered taxa. Therefore, further metagenomic approaches are necessary to provide insights into the important roles of the gut microbiome in the metabolism of common kestrels under captive ecology.

## Conclusion

This study revealed that different foods and living environments significantly affected the gut microbiota of rescued wild common kestrels during captivity. These data verified that Proteobacteria and Firmicutes as well as *Lactobacillus* were more sensitive to changes in the environment and food in captivity. The dominant phylum changed from Proteobacteria to Firmicutes, and the dominant genus *Lactobacillus* significantly increased after captivity. Meanwhile, the functions related to glucose metabolism and amino acid metabolism were significantly enhanced after captivity. Therefore, high quality food provided by humans might improve the nutritional metabolism of the gut microbiota of common kestrels. Although gut microbes facilitate the adaption of hosts to environmental changes, some potential health risks associated with changing from natural foods to a high-fat and high-protein diet cannot be ignored, such as decreased nutrient absorption efficiency and increased metabolic burden on the liver. This also suggests that a balance must be sought between the ratio of natural food and artificial food in the dietary management of rescued wild animals. This could potentially narrow the gap between wild and captive conditions in terms of dietary composition and subsequently ensure a successful return to nature for the rescued animals. Finally, for the rescued common kestrel individuals of different ages, developmental stages, and genders, the possible impact of cage rearing on the composition and abundance of gut microbiota is also a topic worthy of future exploration.

## Data Availability Statement

The datasets presented in this study can be found in NCBI Sequence Read Archive (SRA), PRJNA797889.

## Ethics Statement

The studies involving human participants were reviewed and approved by Animal Ethics Committee, Beijing Forestry University, Beijing, China. Written informed consent for participation was not required for this study in accordance with the national legislation and the institutional requirements.

## Author Contributions

KZ, JS, and XW conceived and designed the experiments. KZ and XG conducted the experiments. KZ and JS analyzed the data and wrote the manuscript. All authors contributed to the article and approved the submitted version.

## Conflict of Interest

The authors declare that the research was conducted in the absence of any commercial or financial relationships that could be construed as a potential conflict of interest.

## Publisher’s Note

All claims expressed in this article are solely those of the authors and do not necessarily represent those of their affiliated organizations, or those of the publisher, the editors and the reviewers. Any product that may be evaluated in this article, or claim that may be made by its manufacturer, is not guaranteed or endorsed by the publisher.

## Footnotes

1
www.biocloud.net
