# Supplementary figures and images for: Gut Microbiome Differences in Rescued Common Kestrels (Falco tinnunculus) Before and After Captivity
Source: Front Microbiol. 2022 Jun 20;13:858592. doi: 10.3389/fmicb.2022.858592 (PMC9251364; doi:10.3389/fmicb.2022.858592)

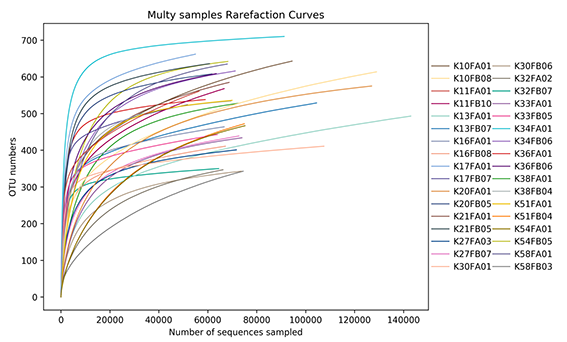

Supplement: Supplementary file 1 [file Image_1.TIFF]

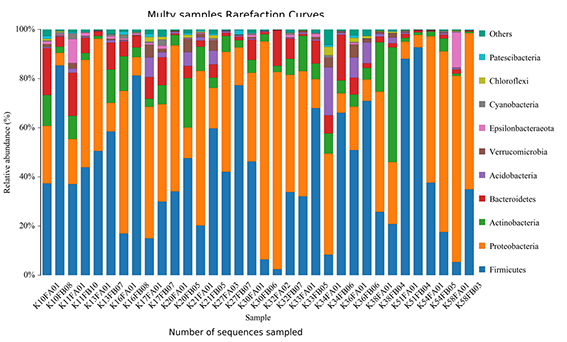

Supplement: Supplementary file 2 [file Image_2.TIFF]
