# Supplementary material for: Gut Microbiome Differences in Rescued Common Kestrels (Falco tinnunculus) Before and After Captivity
Source: Front Microbiol. 2022 Jun 20;13:858592. doi: 10.3389/fmicb.2022.858592 (PMC9251364; doi:10.3389/fmicb.2022.858592)
Supplement: Supplementary file 3 [file Data_Sheet_1.docx]

**Supplementary Table 1** Individual information of the common kestrels tested

| Individual ID | Gender | Age | Rescue location |
| --- | --- | --- | --- |
| K10 | Male | Juvenal | Yanshan Forest Police Station, Fangshan District, Beijing |
| K11 | Male | Juvenal | North Exit, Central District, Pinggu District, Beijing |
| K13 | Male | Adult | Beizang Village Police Station, Daxing District, Beijing |
| K16 | Male | Subadult | Fengtai District Forest Police Station |
| K17 | Female | Adult | Yongshun Town, Tongzhou District, Beijing |
| K20 | Female | Juvenal | Meilian Zhonghe Animal Hospital, Tongzhou District, Beijing |
| K21 | Male | Juvenal | Meilian Zhonghe Animal Hospital, Tongzhou District, Beijing |
| K27 | Female | Adult | Huairou District Miaocheng Police Station |
| K30 | Female | Subadult | Fengyi Police Station, Fengtai District |
| K32 | Male | Adult | No.2 Yard, Anshun Road, Tongzhou District |
| K33 | Female | Subadult | Dongxiaokou Town, Changping District |
| K34 | Male | Juvenal | Heizhuanghu Village, Chaoyang District |
| K36 | Female | Subadult | Runjing Park, Banxi Road, Tongzhou District, Beijing |
| K38 | Female | Subadult | Fangshan Landscaping Bureau |
| K51 | Female | Subadult | Yacui New Village, Yacun Town, Changping District |
| K54 | Male | Juvenal | Laiguangying Police Station, Chaoyang District, Beijing |
| K58 | Female | Adult | Beijing Daxing Airport |

**Supplementary Table 2** Sample collection information.

| Individual  ID | Sample  ID | Environment | Cage time  (day) |
| --- | --- | --- | --- |
| K10 | K10FA01 | Before | — |
| K10 | K10FB08 | After | 14 |
| K11 | K11FA01 | Before | — |
| K11 | K11FB10 | After | 10 |
| K13 | K13FA01 | Before | — |
| K13 | K13FB07 | After | 10 |
| K16 | K16FA01 | Before | — |
| K16 | K16FB08 | After | 9 |
| K17 | K17FA01 | Before | — |
| K17 | K17FB07 | After | 7 |
| K20 | K20FA01 | Before | — |
| K20 | K20FB05 | After | 6 |
| K21 | K21FA01 | Before | — |
| K21 | K21FB05 | After | 6 |
| K27 | K27FA03 | Before | — |
| K27 | K27FB07 | After | 5 |
| K30 | K30FA01 | Before | — |
| K30 | K30FB06 | After | 5 |
| K32 | K32FA02 | Before | — |
| K32 | K32FB07 | After | 4 |
| K33 | K33FA01 | Before | — |
| K33 | K33FB05 | After | 5 |
| K34 | K34FA01 | Before | — |
| K34 | K34FB06 | After | 5 |
| K36 | K36FA01 | Before | — |
| K36 | K36FB06 | After | 5 |
| K38 | K38FA01 | Before | — |
| K38 | K38FB04 | After | 5 |
| K51 | K51FA01 | Before | — |
| K51 | K51FB04 | After | 5 |
| K54 | K54FA01 | Before | — |
| K54 | K54FB05 | After | 5 |
| K58 | K58FA01 | Before | — |
| K58 | K58FB03 | After | 4 |

**Supplementary Table 3** Microbial community node parameters

| Environment | Modularity | ID | Phyla | Degree | Weighted | Closness  centrality |
| --- | --- | --- | --- | --- | --- | --- |
| Before | 0 | Acinetobacter | Proteobacteria | 1 | 0.708768 | 1 |
| Before | 0 | Glutamicibacter | Actinobacteria | 1 | 0.708768 | 1 |
| Before | 1 | Actinomyces | Actinobacteria | 1 | 0.710784 | 1 |
| Before | 1 | Peptoniphilus | Firmicutes | 1 | 0.710784 | 1 |
| Before | 2 | Ruminococcaceae_UCG-014 | Firmicutes | 33 | 27.58882 | 0.492307692 |
| Before | 2 | uncultured_bacterium_f_Muribaculaceae | Bacteroidetes | 32 | 26.87702 | 0.484848485 |
| Before | 2 | Alistipes | Bacteroidetes | 31 | 25.49726 | 0.484848485 |
| Before | 2 | Bifidobacterium | Actinobacteria | 29 | 23.66592 | 0.47761194 |
| Before | 2 | Brevibacterium | Actinobacteria | 29 | 24.15054 | 0.453900709 |
| Before | 2 | Prevotellaceae_UCG-001 | Bacteroidetes | 28 | 22.68312 | 0.460431655 |
| Before | 2 | Akkermansia | Verrucomicrobia | 26 | 21.5095 | 0.444444444 |
| Before | 2 | Bacteroides | Bacteroidetes | 26 | 21.23253 | 0.492307692 |
| Before | 2 | Lactobacillus | Firmicutes | 25 | 19.85711 | 0.42384106 |
| Before | 2 | Streptococcus | Firmicutes | 25 | 20.21451 | 0.429530201 |
| Before | 2 | Desulfovibrio | Proteobacteria | 23 | 18.38205 | 0.447552448 |
| Before | 2 | Corynebacterium_1 | Actinobacteria | 22 | 18.25414 | 0.429530201 |
| Before | 2 | Brachybacterium | Actinobacteria | 21 | 17.10832 | 0.429530201 |
| Before | 2 | Faecalibacterium | Firmicutes | 21 | 16.98574 | 0.412903226 |
| Before | 2 | Ochrobactrum | Proteobacteria | 21 | 16.57321 | 0.429530201 |
| Before | 2 | Leucobacter | Actinobacteria | 19 | 14.34804 | 0.438356164 |
| Before | 2 | Phascolarctobacterium | Firmicutes | 15 | 11.72844 | 0.405063291 |
| Before | 2 | uncultured_bacterium_f_Rhizobiaceae | Proteobacteria | 15 | 11.65122 | 0.463768116 |
| Before | 2 | uncultured_bacterium_o_Chloroplast | Cyanobacteria | 14 | 10.98284 | 0.392638037 |
| Before | 2 | uncultured_bacterium_f_Saccharimonadaceae | Patescibacteria | 12 | 9.02451 | 0.395061728 |
| Before | 2 | Parabacteroides | Bacteroidetes | 10 | 7.509804 | 0.361581921 |
| Before | 2 | uncultured_bacterium_f_Lachnospiraceae | Firmicutes | 7 | 5.122549 | 0.412903226 |
| Before | 2 | Helicobacter | Epsilonbacteraeota | 6 | 4.517157 | 0.367816092 |
| Before | 2 | Campylobacter | Epsilonbacteraeota | 5 | 3.621357 | 0.365714286 |
| Before | 2 | uncultured_bacterium_o_Acidobacteriales | Acidobacteria | 4 | 3.02451 | 0.333333333 |
| Before | 2 | Blautia | Firmicutes | 3 | 2.262255 | 0.335078534 |
| Before | 2 | uncultured_bacterium_f_Ruminococcaceae | Firmicutes | 2 | 1.490196 | 0.31372549 |
| Before | 3 | Alloprevotella | Bacteroidetes | 27 | 22.16584 | 0.453900709 |
| Before | 3 | Capnocytophaga | Bacteroidetes | 27 | 21.64329 | 0.457142857 |
| Before | 3 | Haemophilus | Proteobacteria | 23 | 19.02728 | 0.429530201 |
| Before | 3 | Prevotella_7 | Bacteroidetes | 23 | 18.213 | 0.418300654 |
| Before | 3 | Sulfurovum | Epsilonbacteraeota | 23 | 18.11323 | 0.429530201 |
| Before | 3 | Fusobacterium | Fusobacteria | 21 | 17.07297 | 0.42384106 |
| Before | 3 | Leptotrichia | Fusobacteria | 20 | 16.01087 | 0.421052632 |
| Before | 3 | Neisseria | Proteobacteria | 20 | 16.24882 | 0.421052632 |
| Before | 3 | Lachnospiraceae_NK4A136_group | Firmicutes | 19 | 14.96984 | 0.418300654 |
| Before | 3 | Brevundimonas | Proteobacteria | 18 | 13.70445 | 0.470588235 |
| Before | 3 | Enterorhabdus | Actinobacteria | 17 | 13.44783 | 0.412903226 |
| Before | 3 | Ileibacterium | Firmicutes | 11 | 8.130098 | 0.390243902 |
| Before | 3 | Rothia | Actinobacteria | 8 | 6.122399 | 0.405063291 |
| Before | 3 | Veillonella | Firmicutes | 7 | 5.491881 | 0.345945946 |
| Before | 3 | uncultured_bacterium_f_Sedimenticolaceae | Proteobacteria | 5 | 3.870241 | 0.333333333 |
| Before | 3 | uncultured_bacterium_f_Actinomycetaceae | Actinobacteria | 4 | 3.119448 | 0.402515723 |
| Before | 3 | uncultured_bacterium_f_Coriobacteriales_Incertae_Sedis | Actinobacteria | 2 | 1.567572 | 0.355555556 |
| Before | 4 | Psychrobacter | Proteobacteria | 1 | 0.70098 | 1 |
| Before | 4 | Staphylococcus | Firmicutes | 1 | 0.70098 | 1 |
| Before | 5 | Pseudomonas | Proteobacteria | 1 | 0.806373 | 1 |
| Before | 5 | uncultured_bacterium_f_Enterobacteriaceae | Proteobacteria | 1 | 0.806373 | 1 |
| Before | 6 | Nitrospira | Nitrospirae | 14 | 11.43414 | 0.32 |
| Before | 6 | uncultured_bacterium_f_Blastocatellaceae | Acidobacteria | 14 | 11.31827 | 0.32 |
| Before | 6 | Luteolibacter | Verrucomicrobia | 12 | 9.864 | 0.31372549 |
| Before | 6 | Terrimonas | Bacteroidetes | 12 | 9.883984 | 0.351648352 |
| Before | 6 | uncultured_bacterium_c_Subgroup_6 | Acidobacteria | 12 | 9.773506 | 0.367816092 |
| Before | 6 | uncultured_bacterium_f_Sphingobacteriaceae | Bacteroidetes | 12 | 9.783106 | 0.347826087 |
| Before | 6 | RB41 | Acidobacteria | 11 | 9.155757 | 0.312195122 |
| Before | 6 | uncultured_bacterium_c_Deltaproteobacteria | Proteobacteria | 11 | 9.123541 | 0.312195122 |
| Before | 6 | uncultured_bacterium_o_Rokubacteriales | Rokubacteria | 10 | 8.039905 | 0.310679612 |
| Before | 6 | uncultured_bacterium_f_A4b | Chloroflexi | 9 | 7.37052 | 0.312195122 |
| Before | 6 | uncultured_bacterium_f_Chitinophagaceae | Bacteroidetes | 8 | 6.229303 | 0.280701754 |
| Before | 6 | uncultured_bacterium_f_Gemmatimonadaceae | Gemmatimonadetes | 8 | 6.187875 | 0.292237443 |
| Before | 6 | Bryobacter | Acidobacteria | 6 | 4.840022 | 0.292237443 |
| Before | 6 | MND1 | Proteobacteria | 6 | 4.490393 | 0.271186441 |
| Before | 6 | Allorhizobium-Neorhizobium-Pararhizobium-Rhizobium | Proteobacteria | 5 | 3.80968 | 0.402515723 |
| Before | 6 | Sphingomonas | Proteobacteria | 5 | 4.131772 | 0.277056277 |
| Before | 6 | Bacillus | Firmicutes | 4 | 2.965686 | 0.421052632 |
| Before | 6 | uncultured_bacterium_f_Xanthobacteraceae | Proteobacteria | 4 | 3.051258 | 0.286995516 |
| Before | 6 | uncultured_bacterium_f_Burkholderiaceae | Proteobacteria | 2 | 1.634804 | 0.365714286 |
| Before | 6 | Carnobacterium | Firmicutes | 1 | 0.737953 | 0.219931271 |
| Before | 6 | Rhodanobacter | Proteobacteria | 1 | 0.744474 | 0.219931271 |
| After | 0 | Acinetobacter | Proteobacteria | 1 | 0.735294 | 1 |
| After | 0 | Pseudomonas | Proteobacteria | 1 | 0.735294 | 1 |
| After | 1 | Lachnospiraceae_NK4A136_group | Firmicutes | 30 | 24.84187 | 0.41958042 |
| After | 1 | Bacteroides | Bacteroidetes | 29 | 22.8444 | 0.41958042 |
| After | 1 | Desulfovibrio | Proteobacteria | 29 | 23.54866 | 0.372670807 |
| After | 1 | Alistipes | Bacteroidetes | 22 | 16.96132 | 0.359281437 |
| After | 1 | uncultured_bacterium_f_Muribaculaceae | Bacteroidetes | 13 | 10.46213 | 0.335195531 |
| After | 1 | uncultured_bacterium_o_Chloroplast | Cyanobacteria | 10 | 7.272492 | 0.37037037 |
| After | 1 | uncultured_bacterium_f_Lachnospiraceae | Firmicutes | 6 | 4.52446 | 0.327868852 |
| After | 1 | Dubosiella | Firmicutes | 5 | 3.941176 | 0.310880829 |
| After | 1 | uncultured_bacterium_f_Ruminococcaceae | Firmicutes | 2 | 1.583333 | 0.25 |
| After | 1 | Bifidobacterium | Actinobacteria | 1 | 0.75 | 0.271493213 |
| After | 1 | uncultured_bacterium_f_Corynebacteriaceae | Actinobacteria | 1 | 0.713235 | 0.200668896 |
| After | 2 | Arcobacter | Epsilonbacteraeota | 37 | 31.24874 | 0.45112782 |
| After | 2 | Neisseria | Proteobacteria | 36 | 30.46323 | 0.447761194 |
| After | 2 | Brevibacterium | Actinobacteria | 34 | 28.67257 | 0.434782609 |
| After | 2 | Haemophilus | Proteobacteria | 34 | 28.94761 | 0.434782609 |
| After | 2 | Sulfurovum | Epsilonbacteraeota | 33 | 28.17624 | 0.428571429 |
| After | 2 | Streptococcus | Firmicutes | 32 | 27.68976 | 0.425531915 |
| After | 2 | Veillonella | Firmicutes | 32 | 26.63213 | 0.425531915 |
| After | 2 | Prevotella_7 | Bacteroidetes | 31 | 26.49268 | 0.422535211 |
| After | 2 | uncultured_bacterium_f_Sedimenticolaceae | Proteobacteria | 31 | 26.12824 | 0.428571429 |
| After | 2 | Alloprevotella | Bacteroidetes | 30 | 25.68907 | 0.422535211 |
| After | 2 | Capnocytophaga | Bacteroidetes | 30 | 25.93709 | 0.41958042 |
| After | 2 | Corynebacterium_1 | Actinobacteria | 30 | 23.90542 | 0.41958042 |
| After | 2 | Faecalibacterium | Firmicutes | 30 | 24.96917 | 0.41958042 |
| After | 2 | Fusobacterium | Fusobacteria | 30 | 25.94655 | 0.41958042 |
| After | 2 | Lautropia | Proteobacteria | 30 | 25.48208 | 0.41958042 |
| After | 2 | Brachybacterium | Actinobacteria | 29 | 23.09202 | 0.416666667 |
| After | 2 | Ileibacterium | Firmicutes | 29 | 23.88838 | 0.416666667 |
| After | 2 | Leptotrichia | Fusobacteria | 29 | 23.86261 | 0.416666667 |
| After | 2 | Novosphingobium | Proteobacteria | 29 | 24.49346 | 0.416666667 |
| After | 2 | Parabacteroides | Bacteroidetes | 28 | 22.68964 | 0.413793103 |
| After | 2 | Prevotella | Bacteroidetes | 27 | 22.13132 | 0.46875 |
| After | 2 | Rothia | Actinobacteria | 27 | 22.93328 | 0.472440945 |
| After | 2 | [Eubacterium]_coprostanoligenes_group | Firmicutes | 26 | 22.05848 | 0.408163265 |
| After | 2 | Akkermansia | Verrucomicrobia | 22 | 17.03097 | 0.394736842 |
| After | 2 | Ruminococcaceae_UCG-014 | Firmicutes | 21 | 16.54031 | 0.350877193 |
| After | 2 | Ochrobactrum | Proteobacteria | 19 | 14.27687 | 0.387096774 |
| After | 2 | Leucobacter | Actinobacteria | 15 | 11.41522 | 0.377358491 |
| After | 2 | Blautia | Firmicutes | 14 | 10.48064 | 0.337078652 |
| After | 2 | uncultured_bacterium_f_Rhizobiaceae | Proteobacteria | 12 | 8.842059 | 0.37037037 |
| After | 2 | Bacillus | Firmicutes | 11 | 8.062227 | 0.331491713 |
| After | 2 | uncultured_bacterium_f_Enterobacteriaceae | Proteobacteria | 4 | 2.928676 | 0.317460317 |
| After | 3 | Clostridium_sensu_stricto_1 | Firmicutes | 1 | 0.784314 | 1 |
| After | 3 | Paeniclostridium | Firmicutes | 1 | 0.784314 | 1 |
| After | 4 | Lysinibacillus | Firmicutes | 1 | 0.709015 | 1 |
| After | 4 | Psychrobacter | Proteobacteria | 1 | 0.709015 | 1 |
| After | 5 | RB41 | Acidobacteria | 13 | 9.868447 | 0.294117647 |
| After | 5 | Nitrospira | Nitrospirae | 10 | 8.090868 | 0.28708134 |
| After | 5 | uncultured_bacterium_f_Gemmatimonadaceae | Gemmatimonadetes | 10 | 7.936403 | 0.28708134 |
| After | 5 | uncultured_bacterium_f_A4b | Chloroflexi | 9 | 6.767602 | 0.285714286 |
| After | 5 | Bryobacter | Acidobacteria | 8 | 6.385521 | 0.23715415 |
| After | 5 | MND1 | Proteobacteria | 8 | 6.294338 | 0.236220472 |
| After | 5 | Sphingomonas | Proteobacteria | 8 | 6.184185 | 0.23715415 |
| After | 5 | Luteolibacter | Verrucomicrobia | 7 | 5.793413 | 0.235294118 |
| After | 5 | uncultured_bacterium_c_Subgroup_6 | Acidobacteria | 7 | 5.732552 | 0.235294118 |
| After | 5 | uncultured_bacterium_f_Burkholderiaceae | Proteobacteria | 7 | 5.396187 | 0.236220472 |
| After | 5 | uncultured_bacterium_f_Coriobacteriales_Incertae_Sedis | Actinobacteria | 6 | 4.438897 | 0.350877193 |
| After | 5 | uncultured_bacterium_f_Sphingobacteriaceae | Bacteroidetes | 6 | 4.791004 | 0.234375 |
| After | 5 | Allorhizobium-Neorhizobium-Pararhizobium-Rhizobium | Proteobacteria | 4 | 3.015624 | 0.231660232 |
| After | 5 | uncultured_bacterium_c_Deltaproteobacteria | Proteobacteria | 4 | 3.34442 | 0.229007634 |
| After | 5 | uncultured_bacterium_f_Actinomycetaceae | Actinobacteria | 4 | 3.150406 | 0.279069767 |
| After | 5 | uncultured_bacterium_c_Actinobacteria | Actinobacteria | 3 | 2.171754 | 0.410958904 |
| After | 5 | uncultured_bacterium_f_Chitinophagaceae | Bacteroidetes | 2 | 1.622212 | 0.194174757 |
| After | 5 | uncultured_bacterium_f_Bifidobacteriaceae | Actinobacteria | 1 | 0.896809 | 0.218978102 |
| After | 5 | uncultured_bacterium_o_Rokubacteriales | Rokubacteria | 1 | 0.705162 | 0.191693291 |

**Supplementary Table 4** The functional composition of gut microbes under different living conditions.

| **Function** | **Before** | **After** | ***P*-value** |
| --- | --- | --- | --- |
| Metabolic pathways | 0.1630±0.0004 | 0.1631±0.0004 | 0.9477 |
| Biosynthesis of secondary metabolites | 0.0740±0.0005 | 0.0736±0.0008 | 0.9729 |
| Biosynthesis of antibiotics | 0.0534±0.0006 | 0.0534±0.0006 | 0.9701 |
| Microbial metabolism in diverse environments | 0.0452±0.0006 | 0.0433±0.0003 | 0.0131* |
| Biosynthesis of amino acids | 0.0336±0.0004 | 0.0336±0.0006 | 0.9444 |
| ABC transporters | 0.0332±0.0010 | 0.0322±0.0006 | 0.3940 |
| Carbon metabolism | 0.0266±0.0001 | 0.0262±0.0001 | 0.0411* |
| Two-component system | 0.0249±0.0008 | 0.0232±0.0008 | 0.1400 |
| Purine metabolism | 0.0202±0.0004 | 0.0224±0.0005 | 0.0025** |
| Ribosome | 0.0197±0.0006 | 0.0228±0.0008 | 0.0040** |
| Pyrimidine metabolism | 0.0154±0.0004 | 0.0175±0.0005 | 0.0025** |
| Quorum sensing | 0.0133±0.0003 | 0.0136±0.0003 | 0.4040 |
| Amino sugar and nucleotide sugar metabolism | 0.0104±0.0004 | 0.0127±0.0006 | 0.0047** |
| Pyruvate metabolism | 0.0111±0.0001 | 0.0113±0.0002 | 0.0987 |
| Glycolysis / Gluconeogenesis | 0.0100±0.0002 | 0.0117±0.0004 | 0.0033** |
| Oxidative phosphorylation | 0.0104±0.0004 | 0.0097±0.0004 | 0.2132 |
| Aminoacyl-tRNA biosynthesis | 0.0092±0.0003 | 0.0105±0.0004 | 0.0067** |
| Cysteine and methionine metabolism | 0.0091±0.0001 | 0.0093±0.0002 | 0.6098 |
| Alanine, aspartate and glutamate metabolism | 0.0080±0.0001 | 0.0085±0.0002 | 0.0215* |
| Carbon fixation pathways in prokaryotes | 0.0084±0.0002 | 0.0080±0.0002 | 0.0730 |
| Homologous recombination | 0.0077±0.0002 | 0.0087±0.0002 | 0.0022** |
| Glycine, serine and threonine metabolism | 0.0076±0.0002 | 0.0073±0.0002 | 0.1779 |
| Pentose phosphate pathway | 0.0070±0.0002 | 0.0079±0.0002 | 0.0016 |
| Glyoxylate and dicarboxylate metabolism | 0.0077±0.0003 | 0.0067±0.0003 | 0.0158* |
| Mismatch repair | 0.0066±0.0002 | 0.0076±0.0002 | 0.0060** |
| Bacterial secretion system | 0.0072±0.0002 | 0.0067±0.0003 | 0.2061 |
| Starch and sucrose metabolism | 0.0066±0.0004 | 0.0074±0.0003 | 0.1055 |
| Propanoate metabolism | 0.0071±0.0002 | 0.0067±0.0001 | 0.0916 |
| Peptidoglycan biosynthesis | 0.0066±0.0002 | 0.0073±0.0002 | 0.0160* |
| Fructose and mannose metabolism | 0.0064±0.0004 | 0.0072±0.0003 | 0.1139 |
| Others | 0.3302±0.0020 | 0.3201±0.0024 | 0.0028** |

**Supplementary Table 5** Effects of captivity environment on gut microbes’ alpha diversity index in different age and sex groups of the common kestrel

| **Group** | | **index** | ***t*** | ***df*** | ***W*** | ***P*** |
| --- | --- | --- | --- | --- | --- | --- |
| Sex | Male | ACE | -0.017 | 13.977 | − | 0.987 |
|  |  | Chao1 | -0.342 | 13.822 | − | 0.738 |
|  |  | Simpson | − | − | 16 | 0.844 |
|  |  | Shannon | -0.119 | 10.567 | − | 0.908 |
|  | Female | ACE | 0.196 | 15.16 | − | 0.847 |
|  |  | Chao1 | -0.180 | 15.995 | − | 0.860 |
|  |  | Simpson | − | − | 34 | 0.203 |
|  |  | Shannon | 0.707 | 15.931 | − | 0.489 |
| Age | Juvenal | ACE | 0.064 | 9.769 | − | 0.950 |
|  |  | Chao1 | -0.132 | 7.289 | − | 0.898 |
|  |  | Simpson | − | − | 11 | 1 |
|  |  | Shannon | -0.333 | 7.983 | − | 0.748 |
|  | Subadult | ACE | 0.298 | 6.813 | − | 0.774 |
|  |  | Chao1 | 0.635 | 6.603 | − | 0.547 |
|  |  | Simpson | 0.159 | 9.926 | − | 0.877 |
|  |  | Shannon | -0.020 | 9.358 | − | 0.984 |
|  | Adult | ACE | -0.077 | 7.949 | − | 0.941 |
|  |  | Chao1 | -0.863 | 7.349 | − | 0.415 |
|  |  | Simpson | − | − | 14 | 0.125 |
|  |  | Shannon | 1.147 | 7.865 | − | 0.285 |

**Supplementary Table 6** Effects of captivity environment on gut microbes’ beta diversity index in different age and sex groups of the common kestrel

| **Group** | | **Phylum** | | | **Genus** | | |
| --- | --- | --- | --- | --- | --- | --- | --- |
|  |  | *F* | *R^2^* | *P* | *F* | *R^2^* | *P* |
| Sex | Male | 4.061 | 0.225 | 0.02* | 1.959 | 0.123 | 0.085 |
|  | Female | 0.904 | 0.053 | 0.433 | 1.164 | 0.068 | 0.304 |
| Age | Juvenal | 2.339 | 0.190 | 0.082 | 1.463 | 0.128 | 0.218 |
|  | Subadult | 0.637 | 0.060 | 0.537 | 0.661 | 0.062 | 0.657 |
|  | Adult | 2.567 | 0.243 | 0.119 | 1.840 | 0.187 | 0.113 |

**Supplementary Table 7** Effects of captivity environment on gut microbes’ abundance of dominant phyla and genus in different age and sex groups of the common kestrel

| **Group** | | **Taxonomy** | | ***t*** | ***df*** | ***W*** | ***P*** |
| --- | --- | --- | --- | --- | --- | --- | --- |
| Sex | Male | Phylum | Firmicutes | 1.691 | 12.832 | − | 0.115 |
|  |  |  | Proteobacteria | -2.476 | 13.969 | − | 0.027* |
|  |  |  | Actinobacteria | − | − | 11 | 0.383 |
|  |  |  | Bacteroidetes | -0.288 | 12.847 | − | 0.778 |
|  |  |  | Acidobacteria | − | − | 19 | 0.945 |
|  |  | Genus | *Escherichia-Shigella* | − | − | 18 | 1 |
|  |  |  | *Clostridium_sensu_stricto_*1 | − | − | 22 | 0.641 |
|  |  |  | *Lactobacillus* | 2.409 | 11.742 | − | 0.033* |
|  |  |  | *Paeniclostridium* | − | − | 18 | 1 |
|  |  |  | *Oceanisphaera* | − | − | 5 | 1 |
|  |  |  | *Psychrobacter* | − | − | 7 | 0.148 |
|  |  |  | *Rhodanobacter* | − | − | 16.5 | 0.889 |
|  |  |  | *Carnobacterium* | − | − | 11 | 1 |
|  |  |  | *Romboutsia* | − | − | 17 | 0.945 |
|  |  |  | *Acinetobacter* | − | − | 11 | 0.383 |
|  | Female | Phylum | Firmicutes | 0.412 | 15.806 | − | 0.686 |
|  |  |  | Proteobacteria | -1.473 | 15.987 | − | 0.160 |
|  |  |  | Actinobacteria | − | − | 22 | 1 |
|  |  |  | Bacteroidetes | − | − | 20 | 0.820 |
|  |  |  | Acidobacteria | − | − | 20 | 0.910 |
|  |  | Genus | *Escherichia-Shigella* | − | − | 20 | 0.820 |
|  |  |  | *Clostridium_sensu_stricto_*1 | − | − | 17 | 0.570 |
|  |  |  | *Lactobacillus* | − | − | 37 | 0.098 |
|  |  |  | *Paeniclostridium* | − | − | 14 | 0.359 |
|  |  |  | *Oceanisphaera* | − | − | 12 | 0.799 |
|  |  |  | *Psychrobacter* | − | − | 18 | 0.652 |
|  |  |  | *Rhodanobacter* | − | − | 33 | 0.25 |
|  |  |  | *Carnobacterium* | − | − | 16 | 0.496 |
|  |  |  | *Romboutsia* | − | − | 19 | 0.734 |
|  |  |  | *Acinetobacter* | − | − | 22 | 1 |
| Age | Juvenal | Phylum | Firmicutes | − | − | 16 | 0.313 |
|  |  |  | Proteobacteria | -1.768 | 9.562 | − | 0.109 |
|  |  |  | Actinobacteria | -0.875 | 9.805 | − | 0.402 |
|  |  |  | Bacteroidetes | -0.366 | 8.368 | − | 0.724 |
|  |  |  | Acidobacteria | − | − | 11 | 1 |
|  |  | Genus | *Escherichia-Shigella* |  |  | 8 | 0.688 |
|  |  |  | *Clostridium_sensu_stricto_*1 |  |  | 10 | 1 |
|  |  |  | *Lactobacillus* |  |  | 17 | 0.219 |
|  |  |  | *Paeniclostridium* |  |  | 9 | 0.844 |
|  |  |  | *Oceanisphaera* |  |  | 6 | 0.855 |
|  |  |  | *Psychrobacter* |  |  | 8 | 0.688 |
|  |  |  | *Rhodanobacter* |  |  | 11 | 1 |
|  |  |  | *Carnobacterium* |  |  | 5 | 0.590 |
|  |  |  | *Romboutsia* |  |  | 5 | 0.313 |
|  |  |  | *Acinetobacter* |  |  | 15 | 0.438 |
|  | Subadult | Phylum | Firmicutes | 0.481 | 9.489 |  | 0.642 |
|  |  |  | Proteobacteria |  |  | 4 | 0.219 |
|  |  |  | Actinobacteria |  |  | 7 | 0.563 |
|  |  |  | Bacteroidetes |  |  | 5 | 0.313 |
|  |  |  | Acidobacteria |  |  | 11 | 1 |
|  |  | Genus | *Escherichia-Shigella* |  |  | 13 | 0.688 |
|  |  |  | *Clostridium_sensu_stricto_*1 |  |  | 11 | 1 |
|  |  |  | *Lactobacillus* |  |  | 15 | 0.438 |
|  |  |  | *Paeniclostridium* |  |  | 9 | 0.844 |
|  |  |  | *Oceanisphaera* |  |  | 3 | 0.584 |
|  |  |  | *Psychrobacter* |  |  | 8 | 0.688 |
|  |  |  | *Rhodanobacter* |  |  | 15 | 0.438 |
|  |  |  | *Carnobacterium* |  |  | 16 | 0.313 |
|  |  |  | *Romboutsia* |  |  | 6 | 0.438 |
|  |  |  | *Acinetobacter* |  |  | 8 | 0.688 |
|  | Adult | Phylum | Firmicutes | 0.798 | 7.202 |  | 0.450 |
|  |  |  | Proteobacteria | -2.405 | 7.991 |  | 0.043 |
|  |  |  | Actinobacteria |  |  | 11 | 0.438 |
|  |  |  | Bacteroidetes | 0.372 | 7.636 |  | 0.720 |
|  |  |  | Acidobacteria |  |  | 6 | 0.813 |
|  |  | Genus | *Escherichia-Shigella* |  |  | 6 | 0.813 |
|  |  |  | *Clostridium_sensu_stricto_*1 |  |  | 10 | 0.625 |
|  |  |  | *Lactobacillus* |  |  | 15 | 0.063 |
|  |  |  | *Paeniclostridium* |  |  | 4 | 0.438 |
|  |  |  | *Oceanisphaera* |  |  | 3 | 1 |
|  |  |  | *Psychrobacter* |  |  | 3 | 0.313 |
|  |  |  | *Rhodanobacter* |  |  | 8 | 1 |
|  |  |  | *Carnobacterium* |  |  | 2 | 0.361 |
|  |  |  | *Romboutsia* |  |  | 13 | 0.188 |
|  |  |  | *Acinetobacter* |  |  | 6 | 0.222 |
